# Supplementary material for: Characterizing Strategy Use During the Performance of Hippocampal-Dependent Tasks
Source: Front Psychol. 2020 Sep 1;11:2119. doi: 10.3389/fpsyg.2020.02119 (PMC7490521; doi:10.3389/fpsyg.2020.02119)
Supplement: Supplementary file 1 [file Data_Sheet_1.DOCX]

***Supplementary Material***

**Strategies for each task**

The specific strategies presented to the participants for each task are shown below. Note that for each task the participants were presented only with a list of the strategies and were not aware of the strategy categories. The strategy category information is included to show how the strategies were allocated into the three primary categories for data analysis.

Participants were asked to indicate the use of each strategy by “Yes” or “No”. They could select as many strategies as were relevant to how they performed a task. Half of the participants saw the visual imagery strategies first, the other half saw the verbal strategies first.

For all tasks, the option “Other”, with space to describe new strategies, was also available.

**Scene construction task**

| **Strategy category** |  |
| --- | --- |
| **Scene visual imagery** | I had an immediate visual image of the scene in my mind, with details appearing all at once as a coherent whole. It almost felt like I was really there.  I had a visual image of the scene which I imagined in a piece-by-piece manner, adding details to gradually form a coherent whole.  I had a static 2D visual image of the scene in my mind, like looking at a photograph.  When describing part of the scene to the experimenter, my visual focus was only on that specific section and I did not visualise the rest of the scene around it.  I had a very vague or fleeting sense of a visual image in my mind, which was really unclear and hazy – it was more the idea of an image in my mind than actually seeing an image itself clearly. My very vague impression is that the image was scene-like (in that I had a sense of a space or context, albeit very vague). |
| **Other visual imagery** | I had separate visual images of individual objects I would typically expect to find in the scene, but did not picture the scene as a coherent whole with all the objects together.  I visualised words for possible objects that I would expect to find in the scene as though written in the air.  I visualised sentences that describe the scene, as though written in the air.  I had a very vague or fleeting sense of a visual image in my mind, which was really unclear and hazy – it was more the idea of an image in my mind than actually seeing an image itself clearly. My very vague impression is that the image (choose one):  - had multiple elements but was not a scene  - involved single isolated objects  - I cannot describe the image |
| **Verbal** | I verbally thought about the scene overall, in words or sentences alone, so that the description of the scene I gave was a continuous narrative describing the scene as a coherent whole. I did this without involving visual imagery.  I verbally thought about the scene in sections, using words or sentences alone, so that I built up an overall description gradually piece-by-piece. There was no visual imagery involved in doing this.  I verbally listed, using words and sentences alone, individual objects that I would expect to typically find in the scene, without combining them into a whole. There was no visual imagery involved in doing this. |
| **Other** | Please describe here other strategies you used that are not listed above. |

**Autobiographical Interview (for all memory ages)**

| **Strategy category** |  |
| --- | --- |
| **Scene visual imagery** | I had an immediate, fully-formed visual image of a scene or scenes from my memory that was quite immersive, like a movie playing out.  I had an immediate, fully-formed visual image of my memory, with details appearing all at once as a coherent scene, as though looking at a photograph.  I reconstructed a visual image of the memory, by adding details in a piece-by-piece manner to gradually form a coherent whole scene.  I had a very vague or fleeting sense of a visual image in my mind, which was really unclear and hazy – it was more the idea of an image in my mind than actually seeing an image itself clearly. My very vague impression is that the image was scene-like (in that I had a sense of a space or context, albeit very vague). |
| **Other visual imagery** | I had separate visual images of various individual features and/or people involved in the memory in isolation, but did not picture them all together within a scene or the wider context of the overall memory.  I focused on one image of a particular feature/person involved in the memory in isolation, and described my memory using that as a reference.  I imagined written in my mind the dates/numbers/short facts in regards to the memory.  I imagined written in my mind sentences that described the memory.  I had a very vague or fleeting sense of a visual image in my mind, which was really unclear and hazy – it was more the idea of an image in my mind than actually seeing an image itself clearly. My very vague impression is that the image (choose one):  - had multiple elements but was not a scene  - involved single isolated objects  - I cannot describe the image |
| **Verbal** | I verbally described, using words or sentences alone, the whole memory as a coherent story, without using visual imagery.  I verbally described, using words or sentences alone, each main element of the memory, and combined the individual elements to form a coherent story without using visual imagery.  I verbally listed, using words or sentences alone, single facts about the memory, although not in a coherent or chronological order.  I thought of the word for a single element, fact or detail in particular that stood out. |
| **Other** | Please describe here other strategies you used that are not listed above. |

**Future thinking task**

| **Strategy category** |  |
| --- | --- |
| **Scene visual imagery** | I had an immediate visual image of the scene in my mind, with details appearing all at once as a coherent whole. It almost felt like I was really there.  I had a visual image of the scene which I imagined in a piece-by-piece manner, adding details to gradually form a coherent whole.  I had a static 2D visual image of the scene in my mind, like looking at a photograph.  When describing part of the scene to the experimenter, my visual focus was only on that specific section and I did not visualise the rest of the scene around it.  I had a very vague or fleeting sense of a visual image in my mind, which was really unclear and hazy – it was more the idea of an image in my mind than actually seeing an image itself clearly. My very vague impression is that the image was scene-like (in that I had a sense of a space or context, albeit very vague). |
| **Other visual imagery** | I had separate visual images of individual objects I would typically expect to find in the scene, but did not picture the scene as a coherent whole with all the objects together.  I visualised words for possible objects that I would expect to find in the scene as though written in the air.  I visualised sentences that describe the scene, as though written in the air.  I had a very vague or fleeting sense of a visual image in my mind, which was really unclear and hazy – it was more the idea of an image in my mind than actually seeing an image itself clearly. My very vague impression is that the image (choose one):  - had multiple elements but was not a scene  - involved single isolated objects  - I cannot describe the image |
| **Verbal** | I verbally thought about the scene overall, in words or sentences alone, so that the description of the scene I gave was a continuous narrative describing the scene as a coherent whole. I did this without involving visual imagery.  I verbally thought about the scene in sections, using words or sentences alone, so that I built up an overall description gradually piece-by-piece. There was no visual imagery involved in doing this.  I verbally listed, using words and sentences alone, individual objects that I would expect to typically find in the scene, without combining them into a whole. There was no visual imagery involved in doing this. |
| **Other** | Please describe here other strategies you used that are not listed above. |

**Navigation learning**

| **Strategy category** |  |
| --- | --- |
| **Scene visual imagery** | I created a visual image of the overall layout of the town over the course of watching the movies, as though having a bird’s eye view looking down at the whole town with both routes combined.  I replayed the movies of the routes in my mind, and combined the routes to create an overall visual layout in my mind’s eye.  I replayed in my mind’s eye the movies of each route separately, but could not picture how they crossed over.  I visualised in my mind scenes containing landmarks, all connected in the order in which they appear within the overall layout of the town.  I had a very vague or fleeting sense of a visual image in my mind, which was really unclear and hazy – it was more the idea of an image in my mind than actually seeing an image itself clearly. My very vague impression is that the image was scene-like (in that I had a sense of a space or context, albeit very vague). |
| **Other visual imagery** | I visualised in my mind images of individual landmarks connected in the order in which they appeared, but without visualising other surrounding details such as the background and foreground details, so it was not scene-like.  I had visual images in my mind of individual landmarks, imagining each separately and was not able to connect them together or imagine them within a scene.  I focused on specific features of landmarks (e.g. a shop name, the colour of a building) and tried to form a visual image of them in my mind.  I had a very vague or fleeting sense of a visual image in my mind, which was really unclear and hazy – it was more the idea of an image in my mind than actually seeing an image itself clearly. My very vague impression is that the image (choose one):  - had multiple elements but was not a scene  - involved single isolated objects  - I cannot describe the image |
| **Verbal** | I verbally described to myself, using words or sentences alone, how to navigate from start to finish through the town, combining both routes and describing the relative locations of the landmarks.  I verbally described to myself, using words or sentences alone, how to navigate from start to finish along each route separately, but without combining the routes.  I said to myself, using words or sentences alone, whereabouts along the routes the landmarks occurred (such as the start, middle or end).  I had a sense of the time taken to travel along the route, and the time that passed between landmarks, but this sense of time did not come from visual imagery in my mind.  I verbally noted, using words or sentences alone, the names of individual landmarks and road names, but I could not connect them together.  I verbally noted, using words or sentences alone, particular features of landmarks. |
| **Other** | Please describe here other strategies you used that are not listed above. |

**Navigation movie clip recognition**

| **Strategy category** |  |
| --- | --- |
| **Scene visual imagery** | I created a visual image of the overall layout of the town over the course of watching the movies, as though having a bird’s eye view looking down at the whole town with both routes combined.  I replayed the movies of the routes in my mind, and combined the routes to create an overall visual layout in my mind’s eye.  I replayed in my mind’s eye the movies of each route separately, but could not picture how they crossed over.  I visualised in my mind scenes containing landmarks, all connected in the order in which they appear within the overall layout of the town.  I had a very vague or fleeting sense of a visual image in my mind, which was really unclear and hazy – it was more the idea of an image in my mind than actually seeing an image itself clearly. My very vague impression is that the image was scene-like (in that I had a sense of a space or context, albeit very vague). |
| **Other visual imagery** | I visualised in my mind images of individual landmarks connected in the order in which they appeared, but without visualising other surrounding details such as the background and foreground details, so it was not scene-like.  I had visual images in my mind of individual landmarks, imagining each separately and was not able to connect them together or imagine them within a scene.  I focused on specific features of landmarks (e.g. a shop name, the colour of a building) and tried to form a visual image of them in my mind.  I had a very vague or fleeting sense of a visual image in my mind, which was really unclear and hazy – it was more the idea of an image in my mind than actually seeing an image itself clearly. My very vague impression is that the image (choose one):  - had multiple elements but was not a scene  - involved single isolated objects  - I cannot describe the image |
| **Verbal** | I verbally described to myself, using words or sentences alone, how to navigate from start to finish through the town, combining both routes and describing the relative locations of the landmarks.  I verbally described to myself, using words or sentences alone, how to navigate from start to finish along each route separately, but without combining the routes.  I said to myself, using words or sentences alone, whereabouts along the routes the landmarks occurred (such as the start, middle or end).  I had a sense of the time taken to travel along the route, and the time that passed between landmarks, but this sense of time did not come from visual imagery in my mind.  I verbally noted, using words or sentences alone, the names of individual landmarks and road names, but I could not connect them together.  I verbally noted, using words or sentences alone, particular features of landmarks. |
| **Other** | Please describe here other strategies you used that are not listed above. |

**Navigation scene recognition**

| **Strategy category** |  |
| --- | --- |
| **Scene visual imagery** | I visually replayed parts of the route in my mind to see if the scene on the screen matched any of my mental images of the films.  I had visual mental snapshots of scenes from the routes, in order to see if the scene on the screen matched any of my mental images.  I imagined the movies being replayed on the computer screen, so that the visual image in my mind was of seeing the screen with the movie playing. This helped me to see if I recognised each scene.  I had a very vague or fleeting sense of a visual image in my mind, which was really unclear and hazy – it was more the idea of an image in my mind than actually seeing an image itself clearly. My very vague impression is that the image was scene-like (in that I had a sense of a space or context, albeit very vague). |
| **Other visual imagery** | I had visual images in my mind of individual, isolated landmarks, without visualising other surrounding details such as the background and foreground details, to see if any matched the scene.  I pictured in my mind specific features of isolated landmarks (e.g. a shop name, the colour of a building) to see if they matched the scene.  I had a very vague or fleeting sense of a visual image in my mind, which was really unclear and hazy – it was more the idea of an image in my mind than actually seeing an image itself clearly. My very vague impression is that the image (choose one):  - had multiple elements but was not a scene  - involved single isolated objects  - I cannot describe the image |
| **Verbal** | I verbally described the route to myself, using words or sentences alone, from start to finish to see if the scene matched any point on the route description.  I verbally thought, using words and sentences alone, of the names of individual isolated landmarks and road names, to see if they were in the scene on the screen.  I verbally recalled, using words or sentences alone, particular features of landmarks to see if they were in the scene on the screen. |
| **Other** | Please describe here other strategies you used that are not listed above. |

**Navigation proximity judgements**

| **Strategy category** |  |
| --- | --- |
| **Scene visual imagery** | I had an overall visual mental map of the layout of the town as though having a bird’s eye view looking down at the whole town with both routes combined, and this helped me to judge the proximity of the landmarks.  I replayed the movies of the routes in my mind as though I was re-experiencing the movies and travelling through the town, and this helped me to judge the proximity of the landmarks; I could also visually imagine how the two routes crossed over.  I replayed the movies of each route separately, and could not imagine how they crossed over – so, I could judge distances between landmarks when they were in the same route, but it was more difficult when they were located on different routes.  I visualised mental snapshots of landmarks placed within scenes along the routes to help me to judge the proximity of the landmarks.  I imagined the movies being replayed on the computer screen, so that the visual image in my mind was of seeing the screen with the movie playing. This helped me to judge the proximity of the landmarks.  I had a very vague or fleeting sense of a visual image in my mind, which was really unclear and hazy – it was more the idea of an image in my mind than actually seeing an image itself clearly. My very vague impression is that the image was scene-like (in that I had a sense of a space or context, albeit very vague). |
| **Other visual imagery** | I visualised in my mind images of individual landmarks connected in the order in which they appeared but without visualising other surrounding details such as the background and foreground details. This helped me to judge the proximity of the landmarks.  I had visual images in my mind of isolated and unconnected landmarks, without visualising other surrounding details or in a particular order. This helped me to judge the proximity of the landmarks.  I had a very vague or fleeting sense of a visual image in my mind, which was really unclear and hazy – it was more the idea of an image in my mind than actually seeing an image itself clearly. My very vague impression is that the image (choose one):  - had multiple elements but was not a scene  - involved single isolated objects  - I cannot describe the image |
| **Verbal** | I verbally described, using words or sentences alone, how to navigate from start to finish through the town, combining both routes, to judge distances between the landmarks. There was no visual imagery involved when I did this.  I described to myself, using words or sentences alone, how to navigate from start to finish along each route separately, without combining the routes – so, I could judge distances between landmarks when they were in the same route, but it was more difficult when they were located on different routes. There was no visual imagery involved when I did this.  I verbally described to myself, using words or sentences alone, the relative positions of landmarks to help me to judge the proximity of the landmarks, and I did this without visual imagery.  I had a sense of the time taken to travel along the route and the time that passed between landmarks, and this helped me to judge the proximity of the landmarks. This sense of time did not come from visual imagery in my mind.  I verbally thought, using words or sentences alone, of the names of individual isolated landmarks, and this helped me to do the task.  I verbally recalled, using words or sentences alone, particular features of landmarks, and this helped me to do the task. |
| **Other** | Please describe here other strategies you used that are not listed above. |

**Navigation route knowledge**

| **Strategy category** |  |
| --- | --- |
| **Scene visual imagery** | I had an overall visual mental map of the layout of the town as though having a bird’s eye view looking down at the whole town with both routes combined, in order to identify the correct order of the images.  I replayed the movies of the routes in my mind as though I was re-experiencing the movies and travelling through the town, in order to identify the correct order of the images; I could also visually imagine how the two routes crossed over.  I replayed the movies of each route separately, and could not imagine how they crossed over – so, I could work out the order of landmarks when they were in the same route, but it was more difficult when they were located on different routes.  I visualised mental snapshots of scenes containing landmarks, all in order along the routes, and this helped me to work out the order.  I imagined the movies being replayed on the computer screen, so that the visual image in my mind was of seeing the screen with the movies playing, and this helped me to judge the order of the landmarks.  I had a very vague or fleeting sense of a visual image in my mind, which was really unclear and hazy – it was more the idea of an image in my mind than actually seeing an image itself clearly. My very vague impression is that the image was scene-like (in that I had a sense of a space or context, albeit very vague). |
| **Other visual imagery** | I visualised in my mind images of landmarks connected in the order in which they appeared but without visualising other surrounding details such as the background and foreground details.  I had visual images in my mind of isolated and unconnected landmarks, without visualising other surrounding details, and this helped me to judge the order of the landmarks.  I had a very vague or fleeting sense of a visual image in my mind, which was really unclear and hazy – it was more the idea of an image in my mind than actually seeing an image itself clearly. My very vague impression is that the image (choose one):  - had multiple elements but was not a scene  - involved single isolated objects  - I cannot describe the image |
| **Verbal** | Using words or sentences alone I verbally described how to navigate from start to finish through the town, combining both routes. This helped me to identify the correct order of the images, and I did this without visual imagery.  I described to myself, using words or sentences alone, how to navigate from start to finish along each route separately, without combining the routes – so, I could work out the order of landmarks when they were in the same route, but it was more difficult when they existed in different routes. There was no visual imagery when I did this.  I verbally described to myself, using words or sentences alone, the relative positions of landmarks in order to work out the order of the landmarks. There was no visual imagery when I did this.  I had a sense of the time taken to travel along the route and the time that passed between landmarks, and this helped me to judge the order of the landmarks. This sense of time did not come from visual imagery in my mind.  I verbally thought of the names of individual isolated landmarks using words or sentences alone, which helped me to judge the order of the landmarks.  I verbally recalled, using words or sentences alone, particular features of landmarks, which helped me to judge the order of the landmarks. |
| **Other** | Please describe here other strategies you used that are not listed above. |

**Navigation sketch map**

| **Strategy category** |  |
| --- | --- |
| **Scene visual imagery** | I had an overall visual mental map of the layout of the town as though having a bird’s eye view looking down at the whole town with both routes combined, and drew this mental map.  I replayed the movies of the routes in my mind as though I was re-experiencing the movies and travelling through the town, in order to create an overall mental layout combining both routes and drew this.  I replayed the movies of each route separately and could draw the individual routes, but I struggled to draw how they crossed over.  I visualised mental snapshots of landmarks placed within scenes in order along the routes, and drew the map based upon this.  I imagined the movies being replayed on the computer screen, so that the visual image in my mind was of seeing the screen with the video playing, helping me to draw the sketch map.  I had a very vague or fleeting sense of a visual image in my mind, which was really unclear and hazy – it was more the idea of an image in my mind than actually seeing an image itself clearly. My very vague impression is that the image was scene-like (in that I had a sense of a space or context, albeit very vague). |
| **Other visual imagery** | I visualised in my mind images of landmarks connected in the order in which they appeared to help me draw the sketch map, but without visualising other surrounding details such as the background and foreground details.  I had visual images in my mind of isolated landmarks, imagining each separately and I found it difficult to imagine where they were located on a map in relation to one another.  I imagined specific parts of landmarks in isolation (e.g. a shop name, the colour of a building), and I found it difficult to imagine where they were located on a map.  I had a very vague or fleeting sense of a visual image in my mind, which was really unclear and hazy – it was more the idea of an image in my mind than actually seeing an image itself clearly. My very vague impression is that the image (choose one):  - had multiple elements but was not a scene  - involved single isolated objects  - I cannot describe the image |
| **Verbal** | I verbally described, using words and sentences alone, how to navigate from start to finish through the town, combining both routes in order, and drew the map from this description. I did not have visual imagery when I did this.  I verbally described to myself how to navigate from start to finish along each route separately, using words and sentences alone, and without combining the routes – so, I could draw individual routes, but struggled to draw how they crossed over. I did not have visual imagery when I did this.  I knew factually whereabouts along the routes the landmarks occurred (such as the start, middle or end), and drew the map from there. There was no visual imagery involved when I did this.  I had a sense of the time taken to travel along the route and the time that passed between landmarks, and drew the map from that. This sense of time did not come from visual imagery.  I verbally thought of the names of individual isolated landmarks and road names using words and sentences alone, but I found it difficult to imagine where they were located on a map.  I verbally recalled particular features of landmarks using words or sentences alone, and used this to help with the sketch map. |
| **Other** | Please describe here other strategies you used that are not listed above. |

**Concrete verbal paired associates learning**

| **Strategy category** |  |
| --- | --- |
| **Scene visual imagery** | I created a single visual image incorporating all/most of the word pairs as they were read out – seeing them all together as objects within a single scene or story.  I used a visual representation of all the words in different locations to associate locations with words.  For each word pair, I had a visual image of the objects within one single scene.  I grouped together multiple word pairs, and I had a visual image of these groupings as objects within scenes.  I imagined a word pair as one single object in a particular setting or scene.  I visualised the word pairs as though written down on a piece of paper or on a computer screen.  I noticed the sounds and syllables in the words and tried to remember them. This then caused me to experience related visual imagery. The visual imagery that was evoked could be described as something scene-like  I had a very vague or fleeting sense of a visual image in my mind, which was really unclear and hazy – it was more the idea of an image in my mind than actually seeing an image itself clearly. My very vague impression is that the image was scene-like (in that I had a sense of a space or context, albeit very vague). |
| **Other visual imagery** | I visually imagined a word pair as one single object to represent the pair in isolation. I did not visualise any other contextual information or background.  I visually imagined many of the word pairs as objects grouped together. I did not have any visual imagery other than the groups of objects, including no background or context.  I imagined each word as a separate object, visualising each object on its own with no background or context and each object was not placed within a scene.  I imagined a word pair as one single object on its own with no background or context and each object was not placed within a scene.  I visualised the word pairs as though they were written in the air.  I visualised the overall form of a word so that I had a visual image of the shape and size of it.  I noticed the sounds and syllables in the words and tried to remember them. This did not elicit any visual imagery The visual imagery that was evoked could be described as something comprising single objects (not a scene).  I had a very vague or fleeting sense of a visual image in my mind, which was really unclear and hazy – it was more the idea of an image in my mind than actually seeing an image itself clearly. My very vague impression is that the image (choose one):  - had multiple elements but was not a scene  - involved single isolated objects  - I cannot describe the image |
| **Verbal** | I verbally created a single story to incorporate all/most of the word pairs. There was no visual imagery involved.  I verbally created a story that grouped multiple word pairs. There was no visual imagery involved.  I verbally created a story or sentence to link the two words in the pair. There was no visual imagery involved.  I listened to each word in turn as it was read out, thinking of each word on its own. There was no visual imagery involved.  I used a single word to link each word pair. There was no visual imagery involved.  I noticed the sounds and syllables in the words and tried to remember them. This did not elicit any visual imagery. |
| **Other** | Please describe here other strategies you used that are not listed above. |

**Concrete verbal paired associates delayed recall**

| **Strategy category** |  |
| --- | --- |
| **Scene visual imagery** | I recalled a single visual image that I had created during learning which incorporated all/most of the word pairs as they were read out – seeing them all together as objects within a single scene or story.  I recalled a visual representation of all the words in different locations that I had created during learning.  For each word pair, I recalled a visual image of the objects within one single scene.  I recalled groups of multiple word pairs in one go, and I had a visual image of each of these groupings as objects within scenes.  I recalled a word pair as one single object in a particular setting or scene.  I recalled visual images of the word as though written down on a piece of paper or on a computer screen.  I recalled the sounds and syllables in the words. This then caused me to experience related visual imagery. The visual imagery that was evoked could be described as something scene-like  I had a very vague or fleeting sense of a visual image in my mind, which was really unclear and hazy – it was more the idea of an image in my mind than actually seeing an image itself clearly. My very vague impression is that the image was scene-like (in that I had a sense of a space or context, albeit very vague). |
| **Other visual imagery** | I recalled a visual image of a word pair as one single object to represent the pair in isolation. I did not visualise any other contextual information or background.  I recalled visual images of many of the word pairs as objects grouped together. I did not have any visual imagery other than the groups of objects, including no background or context.  I recalled a visual image of each word as a separate object, visualising each object on its own with no background or context and each object was not placed within a scene.  I recalled a visual image of a word pair as one single object on its own with no background or context and each object was not placed within a scene.  I recalled the two words in a pair as though they were written in the air.  I visually recalled the overall form and shape of words.  I recalled the sounds and syllables in the words. This then caused me to experience related visual imagery. The visual imagery that was evoked could be described as something comprising single objects (not a scene).  I had a very vague or fleeting sense of a visual image in my mind, which was really unclear and hazy – it was more the idea of an image in my mind than actually seeing an image itself clearly. My very vague impression is that the image (choose one):  - had multiple elements but was not a scene  - involved single isolated objects  - I cannot describe the image |
| **Verbal** | I verbally recalled a single story to incorporate all/most of the word pairs. There was no visual imagery involved.  I verbally recalled a story that grouped multiple word pairs. There was no visual imagery involved.  I verbally recalled a story or sentence that linked the two words in the pair. There was no visual imagery involved.  I verbally recalled of a jumble of single words and tried to match the words up verbally. There was no visual imagery involved.  I verbally recalled a single word that linked each word pair. There was no visual imagery involved.  I recalled the sounds and syllables in the words. This did not elicit any visual imagery. |
| **Other** | Please describe here other strategies you used that are not listed above. |

**Abstract verbal paired associates learning***

| **Strategy category** |  |
| --- | --- |
| **Scene visual imagery** | I created a single visual image incorporating all/most of the word pairs as they were read out – seeing them all together as objects within a single scene or story.  I used a visual representation of all the words in different locations to associate locations with words.  For each word pair, I had a visual image of the objects within one single scene.  I grouped together multiple word pairs, and I had a visual image of these groupings as objects within scenes.  I imagined a word pair as one single object in a particular setting or scene.  I visualised the word pairs as though written down on a piece of paper or on a computer screen.  I noticed the sounds and syllables in the words and tried to remember them. This then caused me to experience related visual imagery. The visual imagery that was evoked could be described as something scene-like.  I had a very vague or fleeting sense of a visual image in my mind, which was really unclear and hazy – it was more the idea of an image in my mind than actually seeing an image itself clearly. My very vague impression is that the image was scene-like (in that I had a sense of a space or context, albeit very vague). |
| **Other visual imagery** | I visually imagined a word pair as one single object to represent the pair in isolation. I did not visualise any other contextual information or background.  I visually imagined many of the word pairs as objects grouped together. I did not have any visual imagery other than the groups of objects, including no background or context.  I imagined each word as a separate object, visualising each object on its own with no background or context and each object was not placed within a scene.  I imagined a word pair as one single object on its own with no background or context and each object was not placed within a scene.  I visualised the word pairs as though they were written in the air.  I visualised the overall form of a word so that I had a visual image of the shape and size of it.  I noticed the sounds and syllables in the words and tried to remember them. This did not elicit any visual imagery The visual imagery that was evoked could be described as something comprising single objects (not a scene).  I had a very vague or fleeting sense of a visual image in my mind, which was really unclear and hazy – it was more the idea of an image in my mind than actually seeing an image itself clearly. My very vague impression is that the image (choose one):  - had multiple elements but was not a scene  - involved single isolated objects  - I cannot describe the image |
| **Verbal** | I verbally created a single story to incorporate all/most of the word pairs. There was no visual imagery involved.  I verbally created a story that grouped multiple word pairs. There was no visual imagery involved.  I verbally created a story or sentence to link the two words in the pair. There was no visual imagery involved.  I listened to each word in turn as it was read out, thinking of each word on its own. There was no visual imagery involved.  I used a single word to link each word pair. There was no visual imagery involved.  I noticed the sounds and syllables in the words and tried to remember them. This did not elicit any visual imagery. |
| **Other** | Please describe here other strategies you used that are not listed above. |

*The abstract verbal paired associates task used very low imagery words (e.g., Concept-Instance) which may seem at odds with some of the visual imagery strategies provided (e.g., “For each word pair, I had a visual image of the objects within one single scene”). However, despite the very low imagery ratings of the words used, some participants were able to create visual images of the abstract word pairs. In addition, the strategies for the concrete and abstract verbal paired associates tasks were designed to be similar to enable direct comparisons between the two tests.

**Abstract verbal paired associates delayed recall***

| **Strategy category** |  |
| --- | --- |
| **Scene visual imagery** | I recalled a single visual image that I had created during learning which incorporated all/most of the word pairs as they were read out – seeing them all together as objects within a single scene or story.  I recalled a visual representation of all the words in different locations that I had created during learning.  For each word pair, I recalled a visual image of the objects within one single scene.  I recalled groups of multiple word pairs in one go, and I had a visual image of each of these groupings as objects within scenes.  I recalled a word pair as one single object in a particular setting or scene.  I recalled visual images of the word as though written down on a piece of paper or on a computer screen.  I recalled the sounds and syllables in the words. This then caused me to experience related visual imagery. The visual imagery that was evoked could be described as something scene-like.  I had a very vague or fleeting sense of a visual image in my mind, which was really unclear and hazy – it was more the idea of an image in my mind than actually seeing an image itself clearly. My very vague impression is that the image was scene-like (in that I had a sense of a space or context, albeit very vague). |
| **Other visual imagery** | I recalled a visual image of a word pair as one single object to represent the pair in isolation. I did not visualise any other contextual information or background.  I recalled visual images of many of the word pairs as objects grouped together. I did not have any visual imagery other than the groups of objects, including no background or context.  I recalled a visual image of each word as a separate object, visualising each object on its own with no background or context and each object was not placed within a scene.  I recalled a visual image of a word pair as one single object on its own with no background or context and each object was not placed within a scene.  I recalled the two words in a pair as though they were written in the air.  I visually recalled the overall form and shape of words.  I recalled the sounds and syllables in the words. This then caused me to experience related visual imagery. The visual imagery that was evoked could be described as something comprising single objects (not a scene).  I had a very vague or fleeting sense of a visual image in my mind, which was really unclear and hazy – it was more the idea of an image in my mind than actually seeing an image itself clearly. My very vague impression is that the image (choose one):  - had multiple elements but was not a scene  - involved single isolated objects  - I cannot describe the image |
| **Verbal** | I verbally recalled a single story to incorporate all/most of the word pairs. There was no visual imagery involved.  I verbally recalled a story that grouped multiple word pairs. There was no visual imagery involved.  I verbally recalled a story or sentence that linked the two words in the pair. There was no visual imagery involved.  I verbally recalled of a jumble of single words and tried to match the words up verbally. There was no visual imagery involved.  I verbally recalled a single word that linked each word pair. There was no visual imagery involved.  I recalled the sounds and syllables in the words. This did not elicit any visual imagery. |
| **Other** | Please describe here other strategies you used that are not listed above. |

*The abstract verbal paired associates task used very low imagery words (e.g., Concept-Instance) which may seem at odds with some of the visual imagery strategies provided (e.g., “For each word pair, I had a visual image of the objects within one single scene”). However, despite the very low imagery ratings of the words used, some participants were able to create visual images of the abstract word pairs. In addition, the strategies for the concrete and abstract verbal paired associates tasks were designed to be similar to enable direct comparisons between the two tests.

**Dead or alive task**

| **Strategy category** |  |
| --- | --- |
| **Scene visual imagery** | I recalled a visual image of a media report featuring the individual within a particular scene or context.  I had a visual image in my mind that depicted a scene from when I heard about or discussed the individual.  I visualised in my mind printed or written information about the individual, such as picturing a page in a newspaper or webpage on a screen, as though it was there in front of me.  I recalled sound snippets or sections of audio from a news item or from a conversation about the individual or from the individual themselves. This then caused me to experience related visual imagery. The visual imagery that was evoked could be described as something scene-like.  I had a very vague or fleeting sense of a visual image in my mind, which was really unclear and hazy – it was more the idea of an image in my mind than actually seeing an image itself clearly. My very vague impression is that the image was scene-like (in that I had a sense of a space or context, albeit very vague). |
| **Other visual imagery** | I had a visual image in my mind of the individual, with no background, context or scene around them.  I imagined separate objects and/or items that I associate with the individual, without imagining these items within a scene.  I visualised in my mind words associated with the individual as though written or printed in the air.  I recalled sound snippets or sections of audio from a news item or from a conversation about the individual or from the individual themselves. This then caused me to experience related visual imagery. The visual imagery that was evoked could be described as something comprising single objects (not a scene).  I had a very vague or fleeting sense of a visual image in my mind, which was really unclear and hazy – it was more the idea of an image in my mind than actually seeing an image itself clearly. My very vague impression is that the image (choose one):  - had multiple elements but was not a scene  - involved single isolated objects  - I cannot describe the image |
| **Verbal** | I verbally thought through a media story about the individual using words or sentences alone, as though retelling the story to myself.  I verbally thought through, using words or sentences alone, a time when I heard about or discussed the individual.  The verbal idea of whether the individual was “dead” or “alive” came to mind immediately without me having to bring to mind further contextual or visual information or images.  I listed single items, objects or facts associated with the individual, using words or sentences alone, to logically work out whether the person was living or dead.  I recalled sound snippets or sections of audio from a news item or from a conversation about the individual or from the individual themselves. This did not elicit any visual imagery. |
| **Other** | Please describe here other strategies you used that are not listed above. |

**“Other” responses**

Other responses were examined to ascertain whether or not the descriptions closely resembled strategies that were already listed and, if this was the case, a strategy was reallocated from Other to the relevant strategy. Here, we detail how many rank 1 Other responses were given for each task, and to which strategy category they were reallocated if that was appropriate (scene visual imagery, other visual imagery, verbal), or whether the Other description contained no additional information that could be used for strategy classification (e.g., “I guessed”; “I imagined how I would feel in that scene”). There was no situation where the Other description referred to a new strategy that was not already represented on the list.

For the **scene construction task**, seven participants provided rank 1 Other responses. Two of these responses contained no additional information that could be used for strategy classification and five were reallocated to scene visual imagery strategies. However, all five participants whose Other responses were reallocated to scene visual imagery strategies already indicated rank 1 scene visual imagery strategies from the list provided.

On the **autobiographical interview**, for childhood autobiographical memories, four participants indicated rank 1 Other responses. Three of these responses contained no additional information that could be used for strategy classification and one was reallocated to a scene visual imagery strategy. However, this participant had already indicated a rank 1 scene visual imagery strategy from the list provided. For teenage autobiographical memories, seven participants indicated rank 1 Other responses. Six of these responses contained no additional information that could be used for strategy classification and one was reallocated to a scene visual imagery strategy. Again, however, this participant had already indicated a rank 1 scene visual imagery strategy from the list provided. For adulthood autobiographical memories, six participants indicated rank 1 Other responses, none of which contained additional information that could be used for strategy classification. For autobiographical memories from the last year, five participants indicated rank 1 Other responses, none of which contained additional information that could be used for strategy classification.

For the **future thinking task**, four participants indicated rank 1 Other responses. Three of these responses contained no additional information that could be used for strategy classification and one was reallocated to a scene visual imagery strategy. However, this participant had already indicated a rank 1 scene visual imagery strategy from the list provided.

For the **navigation tasks**, when viewing the movies while learning, four participants indicated rank 1 Other responses. One of these responses contained no additional information that could be used for strategy classification, one was reallocated to a scene visual imagery strategy, one to other visual imagery strategy and one to a verbal strategy. However, all three participants whose other responses were reallocated had already indicated rank 1 strategies in the corresponding strategy category. For the navigation movie clip recognition task, six participants indicated rank 1 Other responses. Four of these responses contained no additional information that could be used for strategy classification, one was reallocated to a scene visual imagery strategy and one to other visual imagery strategy. Again, both the participants whose strategies were reallocated had already indicated rank 1 strategies in the corresponding strategy category. For the navigation scene recognition task, six participants indicated rank 1 Other responses. Four of these responses contained no additional information that could be used for strategy classification, one was reallocated to a scene visual imagery strategy and one to a verbal strategy. For the participant whose Other response was reallocated to a scene visual imagery strategy, they had already indicated a rank 1 scene visual imagery strategy. However, the participant whose Other response was reallocated to a verbal strategy had not indicated using a rank 1 verbal strategy, even though their Other strategy description did match one of the verbal strategies on the list provided. For the navigation proximity judgements task, five participants indicated rank 1 Other responses. Four of these responses contained no additional information that could be used for strategy classification and one was reallocated to other visual imagery strategy. While this participant had not indicated using a rank 1 other visual imagery strategy, the strategy detailed did match other visual imagery strategy from the strategy list provided. For the navigation route knowledge task, five participants indicated rank 1 Other responses, none of which contained additional information that could be used for strategy classification. For the navigation sketch map, three participants indicated rank 1 Other responses, none of which contained additional information that could be used for strategy classification.

For the **concrete verbal paired associates** (VPA) learning task, seven participants indicated rank 1 Other responses. Three of these responses contained no additional information that could be used for strategy classification and four were reallocated to verbal strategies. However, all four participants whose Other responses were reallocated to verbal strategies already indicated rank 1verbal strategies from the list provided. For the concrete VPA delayed recall task, eight participants indicated rank 1 Other responses. Six of the responses contained no additional information that could be used for strategy classification and two were reallocated to verbal strategies. Again, both participants whose Other responses were reallocated to verbal strategies had already indicated rank 1 verbal strategies from the list provided.

For the **abstract VPA** learning task, 15 participants indicated rank 1 Other responses. Four of these responses contained no additional information that could be used for strategy classification, two were reallocated to scene visual imagery strategies, four to other visual imagery strategies and five to verbal strategies. However, all the participants whose strategies were reallocated had already indicated rank 1 strategies in the corresponding strategy category. For the abstract VPA delayed recall task, 15 participants indicated rank 1 Other responses. Ten of these responses contained no additional information that could be used for strategy classification, two were reallocated to scene visual imagery strategies, two to other visual imagery strategies and one to a verbal strategy. As before, all the participants whose strategies were reallocated had already indicated rank 1 strategies in the corresponding strategy category.

Finally, for the **dead or alive** semantic memory task, two participants indicated rank 1 Other responses. One of these responses contained no additional information that could be used for strategy classification while the other was reallocated to other visual imagery strategy. However, this participant had already indicated using a rank 1 other visual imagery strategy from the list provided.

Overall, therefore, examination of the Other responses resulted in very little additional information not already identified by the strategy questionnaires.
